# The influence of *APOE* and *TOMM40* polymorphisms on hippocampal volume and episodic memory in old age

**DOI:** 10.3389/fnhum.2013.00198

**Published:** 2013-05-22

**Authors:** Beata Ferencz, Erika J. Laukka, Martin Lövdén, Grégoria Kalpouzos, Lina Keller, Caroline Graff, Lars-Olof Wahlund, Laura Fratiglioni, Lars Bäckman

**Affiliations:** ^1^Aging Research Center, Karolinska Institutet and Stockholm UniversityStockholm, Sweden; ^2^Department of Psychology, Lund UniversityLund, Sweden; ^3^KI-Alzheimer Disease Research Center, Karolinska InstitutetStockholm, Sweden; ^4^Department of Geriatrics, Karolinska University HospitalStockholm, Sweden; ^5^Division of Clinical Geriatrics, Karolinska InstitutetStockholm, Sweden

**Keywords:** *APOE*, *TOMM40*, episodic memory, hippocampus, cognitive aging

## Abstract

Mitochondrial dysfunction is implicated in neurodegenerative disorders, such as Alzheimer's disease (AD). Translocase of outer mitochondrial membrane 40 (*TOMM40*) may be influential in this regard by influencing mitochondrial neurotoxicity. Little is known about the influence of the *TOMM40* gene on hippocampal (HC) volume and episodic memory (EM), particularly in healthy older adults. Thus, we sought to discern the influence of *TOMM40* single nucleotide polymorphisms (SNPs), which have previously been associated with medial temporal lobe integrity (rs11556505 and rs2075650), on HC volume and EM. The study sample consisted of individuals from the Swedish National Study on Aging and Care in Kungsholmen (SNAC-K) who were free of dementia and known neurological disorders, and 60–87 years of age (*n* = 424). EM was measured by using a 16-item word list with a 2-min free recall period and delineation of the HC was performed manually. The influence of Apolipoprotein E (*APOE*) and *TOMM40* was assessed by 2 × 2 ANOVAs and partial correlations. There was no effect of *APOE* and *TOMM40* on EM performance and HC volume. However, partial correlations revealed that HC volume was positively associated with free recall performance (*r* = 0.21, *p* < 0.01, *r*^2^ = 0.04). When further stratified for *TOMM40*, the observed association between HC volume and free recall in *APOE* ε4 carriers was present in combination with *TOMM40* rs11556505 any T (*r* = 0.28, *p* < 0.01, *R*^2^ = 0.08) and rs2075650 any G (*r* = 0.28, *p* < 0.01, *R*^2^ = 0.08) “risk” alleles. This pattern might reflect higher reliance on HC volume for adequate EM performance among *APOE* ε4 carriers with additional *TOMM40* “risk” alleles suggesting that the *TOMM40* gene cannot merely be considered a marker of *APOE* genotype. Nevertheless, neither *APOE* nor *TOMM40* influenced HC volume or EM in this population-based sample of cognitively intact individuals over the age of 60.

## Introduction

Although much is known about the clinical manifestations of Alzheimer's disease (AD), the etiology and pathophysiology of this disease still remains to be determined. Mitochondrial dysfunction is becoming increasingly highlighted in both pathological and non-pathological aging (Lin and Beal, 2006; Reddy et al., 2010; Swerdlow, 2011; Ferencz et al., 2012). Previously only considered in unison with *APOE*, due to linkage disequilibrium (LD) between the two genes, *TOMM40* was recently found to independently influence age of onset of AD (Roses et al., 2010; Li et al., 2012). In addition *TOMM40* has been associated with risk of AD (Grupe et al., 2007; Abraham et al., 2008; Harold et al., 2009). However, these findings have not been consistently replicated (Cruchaga et al., 2011; Guerreiro and Hardy, 2012; Jun et al., 2012) and it is unclear if the effect can be attributed to *TOMM40* or *APOE* as the two genes are in strong LD. Moreover, there is limited information on the influence of other *TOMM40* SNPs, besides the rs10524523 poly-T length, on age- and AD-sensitive markers, such as hippocampal (HC) volume and episodic memory (EM). Thus, further research is required in normal old adults before we can tease out the influence of genetic variation in *APOE* and *TOMM40* on HC volume and EM memory, irrespective of dementia.

*TOMM40*, codes for the outer mitochondrial membrane pore subunit (TOM40), which is a pore through which proteins readily enter the mitochondria (Humphries et al., 2005). Known as the “powerhouse” of all cells, mitochondria serve an important function in the central nervous system, providing energy essential for neuronal survival (McBride et al., 2006; Baloyannis, 2011). Not always beneficial to the neurons, an increased net production of reactive oxygen species in mitochondria has been implicated in both pathological and non-pathological aging (Lin and Beal, 2006). Moreover, morphological changes within the mitochondria of the hippocampus have been observed in postmortem AD (Hirai et al., 2001; Baloyannis, 2011). The mitochondrial import channel (TOM40) has been implicated in AD as an important site of amyloid precursor protein (APP) accumulation, which in turn is thought to trigger downstream events leading to an increase in reactive oxygen species (H_2_O_2_) and mitochondrial dysfunction. Interestingly, APP accumulation within the mitochondrial import channels was found across cholinergic, dopaminergic, glutamatergic, and GABAergic neuron types in AD and was more abundant in frontal cortex and the HC (Devi et al., 2006). However, we do not know whether *TOMM40* SNPs influence the channel forming subunit that is implicated as a site where APP accumulates. Nonetheless, it is possible that genetic variation in or close to *TOMM40* changes the function of the TOM40 protein, thereby influencing mitochondrial functioning in areas such as the HC (Ferencz et al., 2012), but this proposed mitochondrial disconnection hypothesis has yet to be confirmed.

Recently, *TOMM40* rs2075650 was associated with AD, and deemed the second most significant AD marker in the *APOE* region (Harold et al., 2009; Feulner et al., 2010). The strong Genome Wide Association Study (GWAS) signal for *TOMM40* rs2075650 is interpreted as an *APOE* hit, due to their strong LD, and often the biological implications are considered to originate from *APOE*. Interestingly, the rs2075650, rs11556505 (*TOMM40*) and rs429358 (*APOE*) haplotype show greater genome wide association with AD than *TOMM40* rs20756505 alone (Potkin et al., 2009). HC atrophy is common, albeit not always present, in the preclinical course of AD (Reitz et al., 2011; Tondelli et al., 2011; Lindberg et al., 2012) and the *APOE* ε 4 allele has been shown to influence HC atrophy in prodromal AD (Jack et al., 2012). *TOMM40* may have an additional influence on this trajectory, as *TOMM40* rs2075650 has been associated with HC integrity (Shen et al., 2010; Bis et al., 2012; Vounou et al., 2012). A recent GWAS on imaging phenotypes by Shen and colleagues (2010) found that both *APOE* and *TOMM40* were associated with multiple imaging phenotypes. Interestingly, *TOMM40* rs2075650 was specifically associated with HC and right amygdala volume, in comparison to the *APOE* ε 4 allele that was linked to volumes throughout the brain including neocortical areas (parietal and temporal cortices) and the hippocampus. Thus, *TOMM40* may have a more specific role within the medial-temporal lobe compared to *APOE*, which may apply primarily to *TOMM40* rs2075650 G, and possibly to rs11556505 T.

It is well-established that HC integrity is pivotal to EM performance (Milner, 1972; Tulving and Markowitsch, 1998; Squire et al., 2004). Whereas the ε 4 allele of the *APOE* gene has been linked to impaired EM performance (Small et al., 2004; Nilsson et al., 2006; De Jager et al., 2012), the influence of *TOMM40* SNPs on memory has rarely been investigated. Yet, the *TOMM40* poly-T length has been found to negatively influence EM (Johnson et al., 2011), but this influence may not be independent of *APOE* (Schiepers et al., 2012), nor always replicable (Cruchaga et al., 2011). Recently, the *TOMM40* poly-T length was found to independently modulate the adverse effects of lorazepam-induced memory impairment in aging (Pomara et al., 2011a). It remains to be determined whether *TOMM40* rs11556505 and rs2075650 SNPs influence EM in normal aging.

In the past years, the impact of genes on brain and cognition has been highlighted (Rasch et al., 2010; Wilson et al., 2011), with increasing focus on the synergistic effects of different single nucleotide polymorphisms (SNPs) (Eichler et al., 2010). Thus, the primary aim of this study was to assess the influence of variations in the *TOMM40* gene on HC volume and EM in old adults (>60) without dementia, taking into account *APOE* genotype status. We analyzed two *TOMM40* SNPs (rs11556505 and rs2075650), which have previously been associated with onset of AD (Grupe et al., 2007; Harold et al., 2009; Potkin et al., 2009; Feulner et al., 2010) and HC integrity (Shen et al., 2010; Bis et al., 2012; Vounou et al., 2012). We hypothesized that these *TOMM40* SNPs have an influence on HC volume and EM performance, irrespective of *APOE* status, with rs2075650 G and rs11556505 T “risk alleles” negatively influencing the dependent variables.

## Materials and methods

### Ethics statement

For the current analyses a subsample of participants from the baseline assessment of the Swedish National Study on Aging and Care in Kungsholmen (SNAC-K) was used. The SNAC-K study complies with the declaration of Helsinki, and has been approved by the ethical committee at Karolinska Institutet. All subjects gave informed consent, and in the case of severe cognitive impairment consent was collected from next-of-kin.

### Study sample

The SNAC-K study commenced in 2001, inviting randomly selected participants from the island of Kungsholmen in central Stockholm to examine medical, psychological, and social aspects of health in late adulthood (Laukka et al., 2013). Three thousand three hundred and sixty three individuals participated in baseline assessments that consisted of neuropsychological testing, medical screening and nurse interviews. The participants were randomly selected from eleven age cohorts: 60, 66, 72, 78, 81, 84, 87, 90, 93, 96, and 99 years and older. For the current analyses, we used a sample of 555 randomly selected non-institutionalized individuals from SNAC-K, who underwent magnetic resonance imaging (MRI). DNA and neuropsychological assessments were available for 496 of these individuals. After exclusions based on the following criteria: age 90 and above (*n* = 14), major depressive episode according to DSM-IV (*n* = 1), MMSE ≤ 24 (*n* = 2), Guillain–Barré syndrome (*n* = 1), epilepsy (*n* = 2), alcohol dependence syndrome (*n* = 3), dyslexia (*n* = 3), stroke (*n* = 19), and suboptimal MRI image quality (*n* = 27), the final study sample comprised 424 individuals between 60 and 87 years of age. The mean age of the participants was 69.91 years (*SD* = 8.63); they had received on average 12.61 years of schooling, had a mean Mini-Mental State Examination score of 29.17 (*SD* = 1.00), and the majority of the sample was female (59.30%).

### MRI acquisition and volumetric measurement

MRI data were acquired on a 1.5 T scanner (Philips Intera, Netherlands). 3D fast field echo (FFE) T1, axial SE (spin echo) Proton Density/T2, axial FLAIR (fluid-attenuated inversion recovery), and axial diffusion tensor imaging data were acquired. The 3D FFE T1 images (*TR* = 15 ms, *TE* = 7 ms, Flip angle = 15°, number of axial slices = 128 with thickness = 1.5 mm and in plane resolution 0.9375 × 0.9375, no gap, Field Of View = 240 × 240, matrix = 196 × 256) were used for volumetry. Volumetric measurements of the HC were performed manually on the HERMES workstation (Nuclear Diagnostics, Stockholm, Sweden) by a radiologist who was blind to the participants' genotype status. The HC formation was defined as gray matter including the HC proper (Ammon's horn), the dentate gyrus, the subiculum and some white matter (alveus, fimbria). The parahippocampal gyrus, entorhinal cortex, and fornix were excluded (Zhang et al., 2010). Intracranial volume (ICV) was acquired using STEREOLOGY (Sheline et al., 1996) in the HERMES workstation and included total brain, dura, ventricles, extraventricular CSF, brain stem, and cerebellum. Intra-rater reliability of 15 randomly selected subjects provided intraclass correlation coefficients above 0.93 for the HC and ICV. Left HC volume (*M* = 3.33 cm^3^, *SD* = 0.50) and right HC volume (*M* = 3.41 cm^3^, *SD* = 0.48) were highly correlated (*r* = 0.83, *p* < 0.00). Thus, volumes from both hemispheres were combined, yielding an overall HC volume of (*M* = 6.74 cm^3^, *SD* = 0.89). We corrected for head size in all analyses by adding ICV as a covariate.

### Neuropsychological assessment

EM was measured using a 16-item word list. Sixteen unrelated concrete nouns were presented to participants both visually and orally. Presentation rate was 5 s per word followed by a 2-min free recall period. We used total number of words recalled as a measure of free recall.

### Genotyping

DNA was extracted from peripheral blood samples using standard methods. Genotyping was performed using MALDI-TOF analysis on the Sequenom MassARRAY^™^ platform at the Mutation Analysis Facility, Karolinska Institutet. DNA was amplified using primer pairs to identify SNP loci, followed by assessing the allele-specific extension products by their respective mass, using MassARRAY MALDI-TOF mass spectrometry. Quality control was performed at the DNA-sample level, assay level, and the level of multiplex assay pool. The call rates for the SNPs included in this study were between 99.4 and 99.7%. Concordance analysis was done on 486 DNA samples that were present twice, and 14 DNA samples that were present in triplets, and analyzed in separate runs. Analysis performed on all assays passing the quality controls, yielded a concordance score >99%.

We targeted two *TOMM40* polymorphisms: rs11556505 and rs2075650 and for *APOE* we assessed the rs429358 SNP that determines ε 4 status. Dichotomized variables were created for *APOE* rs429358 (no ε 4 vs. any ε 4) and *TOMM40* rs11556505 (CC vs. any T) and rs2075650 (AA vs. any G). Allelic frequencies (Table 1) were in line with previous reports and Hardy Weinberg Equilibrium was *p* = 0.83 for rs429358, *p* = 0.01 for rs11556505 and *p* = 0.01 for rs2075650, all considered within the range of established conventions. LD was calculated between *TOMM40* and *APOE* SNPs using the statistical package SNPassoc in R (Gonzalez et al., 2007). In the current sample *TOMM40* SNPs were in LD (*D*′_max_ = 1), with *APOE*. Furthermore, the two *TOMM40* SNPs (rs11556505 and 2075650) were in high LD (*D*′ = 1). However, their frequency was not completely overlapping and, therefore, they were considered separately in the analyses.

**Table 1:** **Polymorphic characteristics of the *APOE* and *TOMM40* gene**.

**SNP**	**Gene**	**Chr**	**Position**	**Frequencies**	**Protein**	**LD rs429358**
rs429358	*APOE*	19	45411941	any ε4 27.2%	Apolipoprotein E	
rs2075650	*TOMM40*	19	45395619	any G 27.9%	Tom40	*D*′ = 0.83
rs11556505	*TOMM40*	19	45396144	any T 27.5%	Tom40	*D*′ = 0.83

LD, Linkage Disequilibrium measured in *D*′; TOMM40, Translocase of outer mitochondrial membrane 40; APOE, Apolipoprotein E; Chr, Chromosome.

### Statistical analyses

We used IBM SPSS 19 for all analyses. Primary analyses to evaluate the genetic influence on EM included a series of analysis of variance (ANOVAs), with dichotomized genotype groups as factors i.e., *APOE* rs429358 (no ε 4 vs. any ε 4) and *TOMM40* rs11556505 (CC vs. any T) and EM free recall as dependent variable. This way both main and interaction effects of *APOE* and *TOMM40* were facilitated. To assess the influence of the *APOE* and *TOMM40* SNPs on HC volume a series of analysis of covariance (ANCOVA) were conducted, with ICV as a covariate. Second order partial correlations, controlling for age and ICV, were conducted in order to assess the association between EM and HC volume. To ascertain if there was a statistical difference between correlation coefficients Fisher's z transformation (Fisher, 1915) was utilized.

## Results

### The influence of APOE and TOMM40 on EM

**Figure 1:**
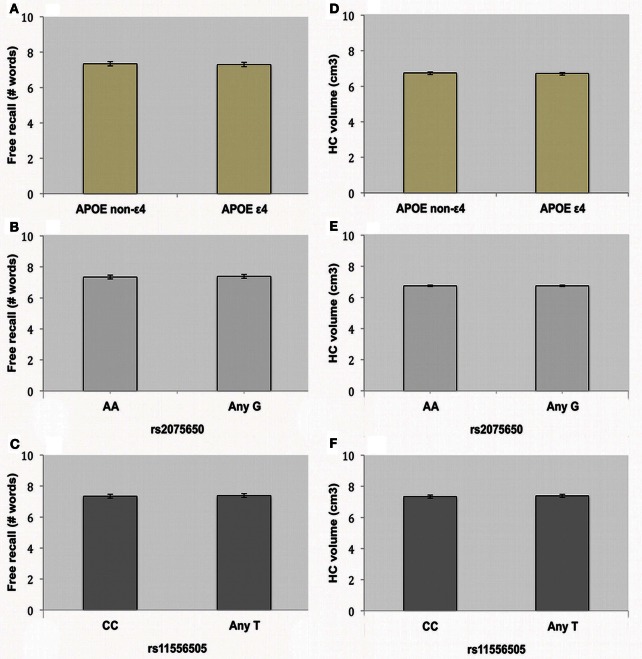
**Influence of the *APOE* and *TOMM40* genes on episodic memory.** Figures depict mean and standard deviation for free recall performance (Number of words) and hippocampal (HC) volume (cm^3^) in *APOE* **(A,D)**, *TOMM40* rs20756505 **(B,E)** and rs11556505 **(C,F)**.

2 × 2 ANOVAs were performed to assess the influence of *APOE* and *TOMM40* on EM free recall, one model per *TOMM40* SNP. Demographic variables are displayed in Table 2 and differences were analyzed using ANOVAs. There was no main effect of the *APOE* polymorphism on free recall performance (Figure 1A), meaning that presence or absence of the ε 4 allele had no influence on EM in the current sample [*F*_(1, 409)_ = 0.49, *p* = 0.486]. Furthermore, neither *TOMM40* SNPs, rs20756505 [*F*_(1, 409)_= 0.29, *p* = 0.589] nor rs11556505 [*F*_(1, 408)_ = 0.29, *p* = 0.589], had a significant influence on free recall performance (Figures 1B,C). Finally, we observed no interaction effects between the *APOE* and *TOMM40* SNPs on the dependent variable free recall (1) rs20756505 [*F*_(1, 409)_= 0.25, *p* = 0.621], (2) rs11556505 [*F*_(1, 408)_= 0.23, *p* = 0.632]. The same pattern was observed that is no main or interaction effects of *APOE* and *TOMM40* SNPs, when age, sex and education were adjusted for using ANCOVAs (*p*s > 0.10).

**Table 2:** **Demographic variables across *APOE* and *TOMM40* status**.

	***APOE* no ε 4**				***APOE* any ε 4**					
***TOMM40***	**M**	**SD**	**M**	**SD**	**M**	**SD**	**M**	**SD**	***F***	***P* value**
rs2075650	AA *n* = 271		Any G *n* = 30		AA *n* = 29		Any G *n* = 84			
Age	69.80	8.5	71.73	9.5	70.69	9.4	69.88	8.9	0.52	0.67
Education	12.57	4.5	11.28	3.5	12.50	4.2	13.27	4.7	1.53	0.21
MMSE	29.24	0.9	28.97	0.9	29.04	1.2	29.02	1.1	1.68	0.17
Sex M/F	108/163		13/17		11/18		33/51		0.21^†^	0.98
rs11556505	CC *n* = 272		Any T *n* = 30		CC *n* = 29		Any T *n* = 82			
Age	69.87	8.5	71.73	9.5	70.69	8.0	69.83	9.0	0.49	0.69
Education	12.57	4.5	11.28	3.5	12.41	4.3	13.31	4.7	1.61	0.19
MMSE	29.24	0.9	28.97	0.9	29.00	1.2	29.03	1.1	1.69	0.17
Sex M/F	109/163		13/17		11/18		33/49		0.19^†^	0.98

† = χ^2^;

MMSE, Mini Mental State Examination;

TOMM40, Translocase of outer mitochondrial membrane 40;

APOE, Apolipoprotein E.

### The influence of APOE and TOMM40 on HC volume

In order to assess the influence of the *APOE* and *TOMM40* SNPs on HC volume we conducted 2 × 2 ANCOVAs, one per *TOMM40* SNP, with HC volume as dependent variable. We included ICV as a covariate in the model to ensure that there were no confounding effects on HC volume as a result of variation in ICV. Running these ANCOVAs with *APOE* (rs429358) and *TOMM40* (rs2075650 and rs11556505) resulted in no significant main effect of *APOE* [*F*_(1, 409)_= 0.58, *p* = 0.447], or *TOMM40* rs20756505 [*F*_(1, 409)_= 0.07, *p* = 0.799], rs11556505 [*F*_(1, 408)_= 0.01 *p* = 0.918] and no interaction effects (1) rs20756505 [*F*_(1, 409)_= 0.54 *p* = 0.462], (2) rs11556505 [*F*_(1, 408)_ = 0.30 *p* = 0.583] (Figures 1D–F). This suggests that neither *APOE* nor *TOMM40* polymorphisms have a significant influence on HC volume in individuals with no known neurodegenerative disorders, age 60–87. Again, no main or interaction effects were observed when controlling also for age, sex and education (*p*s > 0.10).

**Figure 2:**
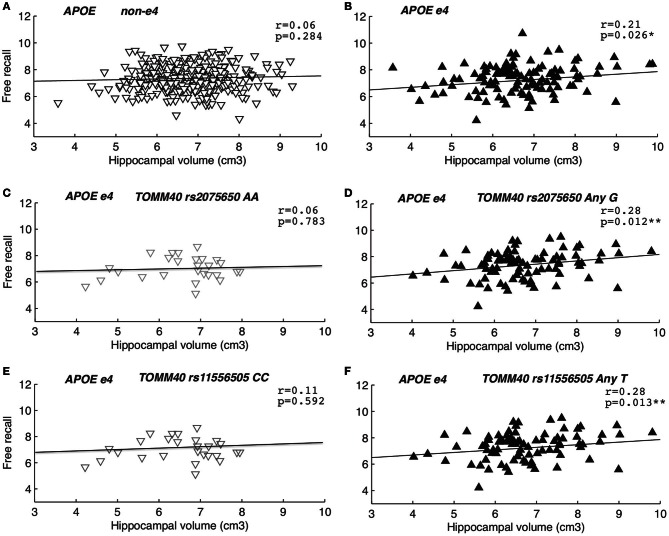
**The association between hippocampal volume and episodic memory.** Second order partial correlations, between free recall performance and hippocampal volume, controlling for age and intracranial volume. Panels **(A,B)** depicts initial partial correlations for *APOE* non-ε 4 vs. ε 4 carriers, followed by partial correlations in *APOE* ε 4 carriers only according to *TOMM40* dichotomization: **(C)** rs2075650 AA, **(D)** rs2075650 any G, **(E)** rs11556505 CC, **(F)** rs11556505 any T.

To test if *TOMM40* genotypes influenced the association between EM and HC volume we conducted a series of partial correlations between free recall and HC volume. All analyses were controlled for age, which is known to influence HC volume in the SNAC-K sample (Zhang et al., 2010), as well as EM (Rönnlund et al., 2005), and ICV (Sanfilipo et al., 2004). The correlations were conducted in two steps; first analyzing the influence of *APOE* (non-ε 4 vs. any ε 4), followed by the influence of *TOMM40* rs2075650 (AA vs. any G) and rs11556505 (CC vs. any T) in *APOE* ε 4 carriers only. We observed a significant correlation between HC volume and free recall performance in *APOE* ε 4 carriers (*r* = 0.21, *p* = 0.026), such that those carrying the ε 4 allele appear to require larger HC volume in order to perform well on free recall (Tulving and Craik, 2000), accounting for 4% of the variance (Figure 2B). This association was not significant in *APOE* non-ε 4 carriers (*r* = 0.06, *p* = 0.284) (Figure 2A), and when tested with the Fisher z-transformation there was a non-significant trend for differences between the correlations for any ε 4 and non-ε 4 carriers (*p* = 0.09). Taking this association one step further, we assessed whether the positive correlation between HC volume and free recall was influenced by the presence or absence of *TOMM40* “risk” alleles in *APOE* ε 4 carriers only (Figures 2D,F). The positive correlation between EM and HC volume was present among *TOMM40* “risk” allele carriers rs2075650 (any G: *r* = 0.28, *p* = 0.012) and *TOMM40* rs11556505 (any T: *r* = 0.28, *p* = 0.013) carriers with any *APOE* ε 4 allele, explaining 8% of the variance, in comparison to “non-risk” allele carriers rs2075650 (AA: *r* = 0.16, *p* = 0.420) and rs11556505 (CC: *r* = 0.19, *p* = 0.338) with an *APOE* ε 4 allele. Moreover, *TOMM40* SNPs had no additional influence on the non-significant association among *APOE* non-ε 4 carriers (*p*s > 0.10). These findings were replicated when also controlling for a sex and education [*APOE* ε4 carriers and TOMM40 rs2075650 (any G: *r*=0.28, *p*=0.012) and TOMM40 rs11556505 (any T: *r*=0.28, *p*=0.012)]. In order to correct for multiple comparisons we utilized Bonferroni correction whereby the conventional level of significance (*p* < 0.05) was divided by the number of SNPs utilized (*n* = 3), yielding a more stringent α-level of *p* < 0.016. Whereas the association between HC volume and free recall performance among *APOE* ε 4 carriers did not survive correction, the association among *APOE* ε 4 carriers with *TOMM40* risk alleles remained significant (Figures 2D,F).

Upon closer examination of the correlation coefficients using Fisher z transformation (Fisher, 1915) we observed that the correlations were not significantly different in *TOMM40* “risk” vs. “non-risk” allele carriers with the presence of an *APOE* ε 4 allele. Removal of one influential case (>2 SD HC volume and EM memory) in APOE ε 4 carriers with an additional *TOMM40* “non-risk” allele (Figures 2C,E), resulted in a reduction of the magnitude of the correlation coefficient from *r* = 0.16 to *r* = 0.06 for rs2075650 (AA) and from *r* = 0.19 to *r* = 0.11 for rs11556505 (CC). Note that data are presented following removal of outliers. Nevertheless, even with this reduced sample, Fishers *z* test (Fisher, 1915) resulted in no significant difference in correlation coefficients across *TOMM40* genotypes (*p*s > 0.10). No other outliers were present in the sample.

Finally, we confirmed the specificity of the association between HC volume and free recall, using a battery of tasks assessing semantic memory, verbal fluency and perceptual speed (Laukka et al., 2013). For none of these task domains, the relationship attained significance (*p*s > 0.10). All findings were replicated following exclusion of participants (*n* = 12) with a preliminary dementia diagnosis at follow-up, which took place 3 years after baseline in the old cohorts (age ≥ 78 at baseline) and 6 years after baseline (age 60–78 at baseline), to ensure that the results were not biased by the presence of preclinical AD cases.

## Discussion

The aim of the current study was to assess whether there is a differential influence of *APOE* and *TOMM40* polymorphisms on HC volume and EM in non-demented older adults. Our findings suggest that the *APOE* and *TOMM40* SNPs examined do not influence HC volume and EM performance in adults above 60 years of age.

Initial analyses revealed that there was no influence of the *APOE* ε 4 allele on free recall performance, which is in line with previous research showing limited influence of *APOE* ε 4 on cognition in individuals free of dementia (Small et al., 2000), especially when accounting for preclinical dementia (Savitz et al., 2006; Laukka et al., 2013). Thus, our results do not provide support for a significant effect of the *APOE* ε 4 allele on HC volume and EM. Nonetheless, the primary aim of the current study was to gauge the influence of *TOMM40* SNPs on free recall and HC volume. Although *TOMM40* rs2075650 has been associated with HC volume in AD (Shen et al., 2010), we observed no association between either *TOMM40* rs11556505 nor rs2075650 on HC volume in this sample of old cognitively normal individuals. Likewise, we did not find an association between *TOMM40* SNPs and EM performance. Thus, it appears that these *TOMM40* SNPs have no influence on HC volume and EM performance in non-demented older adults as measured in this study.

Currently, the majority of research concerning the influence of *TOMM40* on cognition has been focused on the *TOMM40* poly-T repeat (rs10524523). Recently, Hayden and colleagues (2012) observed that *TOMM40* length variation within the rs10524523 SNP influenced cognition in normal aging, even when accounting for *APOE*. In the current sample, neither *TOMM40* polymorphisms (rs2075650, rs11556505) had a direct influence on cognition. Yet it is possible that the differential effect of *APOE* and *TOMM40* is age-dependent and therefore not present in the current sample. A recent longitudinal study by Caselli and colleagues (2012) demonstrated that variation in the *TOMM40* poly-T length influences memory performance independently of *APOE* variation prior to the age of 60. Moreover, our understanding of the biological expression of *TOMM40* SNPs remains limited. Indeed, a recent study by Hedskog and colleagues (2012) found that the *TOMM40* rs10524523 repeat length polymorphism (poly-T), had no detectable influence on mitochondrial morphology when measured in fibroblasts in healthy individuals before 64 years of age. However, there is a lack of knowledge regarding the influence of other *TOMM40* polymorphisms on mitochondrial functioning and morphology in individuals above this age. A recent study by Bekris and colleagues (2012) showed that regions within *TOMM40*, including *TOMM40* poly-T and rs2075650, are involved in *APOE* and *TOMM40* gene regulation by influencing promoter activity in certain haplotype structures depending on haplotype and cell type. These findings suggest that gene expression within the *TOMM40* region is complex and haplotype- as well as cell-dependent. However, despite some implications from the literature of a *TOMM40* component to EM and HC volume, we were not able to confirm this association with the current *TOMM40* SNPs in our population based sample of non-demented older individuals.

Whereas, a direct influence of *TOMM40* variation was not evident in the current study, we observed a significant association between HC volume and free recall performance in *APOE* ε 4 carriers who also carried *TOMM40* “risk alleles.” The relationship between HC volume and EM in *APOE* ε 4 carriers with the two *TOMM40* “risk alleles” were the only correlation that survived correction for multiple testing. Moreover, the association between HC volume and free recall in *APOE* ε 4 carriers with *TOMM40* “non-risk alleles” was markedly reduced upon the removal of an outlier, strengthening our hypothesis that the strongest association is found in *APOE* ε 4 carriers with *TOMM40* “risk alleles.” Although the association was primarily present in *APOE* ε 4 carriers who also carry *TOMM40* “risk alleles” it should be noted that the correlation coefficients in question were not significantly different and the lack of association in *APOE* ε 4 carriers with *TOMM40* “non-risk alleles” may reflect small sample size in this group.

Nevertheless, the positive association between HC volume and free recall performance indicates that *APOE* ε 4 allele carriers who also carry a *TOMM40* “risk allele” may be more dependent on HC volume for proficient EM performance, in comparison to “non-risk” allele carriers of these polymorphisms. Although both *TOMM40* SNPs were driving the association between HC volume and EM memory performance in *APOE* ε 4 carriers, the two *TOMM40* SNPs are in perfect LD. However, the frequencies of TOMM40 SNPs are not completely overlapping.

Our findings indicate that, although variation within the *TOMM40* gene does not directly influence HC volume, the polymorphisms seem to have an effect on HC functioning, beyond that of *APOE* ε 4 alone. This finding lends support to the mitochondrial disconnection hypothesis (Ferencz et al., 2012), suggesting that there may be a mitochondrial component of HC functioning in normal aging, with a possible *TOMM40*-induced oxidative stress in HC neurons that does not influence volume *per se*, but rather the function of this brain structure in old adults. This result is in line with data demonstrating an independent influence of *TOMM40* polymorphisms on markers of neuronal damage. Higher levels of neurofilament light proteins, a sign of neuronal damage, have been found in the cerebrospinal fluid of cognitively intact individuals, but only among *APOE* ε 4 carriers who do not carry the short poly-T variant of *TOMM40*, a variant that is considered protective (Bruno et al., 2012). *APOE* ε 4 alone did not influence markers of neuronal damage suggesting that it is the negative combination of the two genes that is associated with neuronal damage. Although we did not test markers of neuronal damage in our current sample, our findings indicate that *APOE* ε 4 may be potentially detrimental to HC functioning only in combination with *TOMM40* “risk alleles.” This hypothesis will have to be confirmed in future fMRI studies.

Other studies focusing on the *TOMM40* poly-T length have shown that *TOMM40* poly-T length has limited influence on mitochondrial morphology (Hedskog et al., 2012), and levels of amyloid and tau in cerebrospinal fluid (Pomara et al., 2011b), suggesting that *TOMM40*-modulated neurotoxicity may not be markedly present in healthy aging. Thus, in non-pathological aging, where HC volume and EM are relatively intact (Kalpouzos et al., 2009; Nyberg et al., 2012), mitochondrial morphology may be well-preserved among *TOMM40* “risk” allele carriers, in line with the lack of direct influence of *TOMM40* SNPs on medial temporal lobe integrity observed in the current sample. That said, there may already be signs of *TOMM40*-induced neuronal damage in old adults (Bruno et al., 2012), accounting for the added influence of *TOMM40* (rs11556505 any T allele, rs2075650 any G allele) on the association between HC integrity and free recall in *APOE* ε 4 carriers observed here. Future studies in healthy populations taking in consideration markers of neuronal damage and oxidative stress should confirm this hypothesis.

One could argue that the association we observed is primarily dependent on *APOE* ε 4, as *APOE* and the current *TOMM40* SNPs were in LD. However, the presence of an association among *APOE* ε 4 carriers with TOMM40 “risk alleles” speaks against this notion. Thus, despite high LD between *TOMM40* rs20756505 and rs1155650 and *APOE*, there appears to be an added influence of *TOMM40* “risk alleles” on the HC volume—EM link.

### Limitations

Our findings should be considered in light of some limitations. Although we have a large sample, power concerns may still apply with regard to the effects of the specific genes on brain and cognition. *Post-hoc* power calculations were conducted utilizing G^*^Power3 (Faul et al., 2007). Based on the assumption that the effect size for the genetic influence on memory performance and HC volume is η^2^ = 0.1 (Rasch et al., 2010; Laukka et al., 2013), our sample of *n* = 424 individuals provides sufficient, albeit low, power (60%) to detect a significant difference (α = 0.05) in memory performance between genotype groups. In addition, our sample size was too small to examine potential dose-response effect for the *APOE* ε 4 allele. We did not observe any direct influence of *APOE* and *TOMM40* polymorphisms in the current sample. Yet, it has been suggested that, in order to tease out the true effects in this region that is in LD, sample sizes of up to 20 000 would be required (Guerreiro and Hardy, 2012).

There is an inherent limitation in the cross-sectional nature of our study and future studies should assess if *TOMM40* SNPs are associated with longitudinal changes. The current sample was too small to assess the gene-memory link per age-cohort but future studies with larger sample sizes should address this matter. Thus, future longitudinal studies with larger sample sizes should consider the independent influence of *APOE* and *TOMM40* on rate of brain and memory change, and how this relationship is expressed in different age cohorts. Potential confounders that may have influenced our findings include age, sex, and education. However, our findings were replicated after controlling for these confounding factors. Moreover, we limited the potential influence of incipient dementia by replicating our findings following exclusion of preclinical dementia cases, suggesting that incipient dementia had no influence on the current findings.

## Concluding remarks

In conclusion, we found no influence of *APOE* and *TOMM40* polymorphisms on HC volume and EM in old adults suggesting that variation in these genes has limited influence on brain and cognition in non-demented adults above age 60. Our findings, however, provide some, albeit limited, support for an added influence of *TOMM40* polymorphisms (rs2075650, rs11556505) on EM in normal aging. The aforementioned findings of the current study suggest that individuals with the *APOE* ε 4 allele in combination with *TOMM40* rs2075650 and rs11556505 “risk-alleles” may have greater reliance on HC volume for adequate EM performance. Whether the influence of the *TOMM40* gene is more pivotal in prodromal AD remains to be seen and future follow-up examination of the study sample should provide evidence that speaks to this issue.

### Conflict of interest statement

The authors declare that the research was conducted in the absence of any commercial or financial relationships that could be construed as a potential conflict of interest.
